# Overcoming COVID-19 in West African countries: is herd immunity an option?

**DOI:** 10.11604/pamj.supp.2020.35.2.24217

**Published:** 2020-07-06

**Authors:** Olayinka Stephen Ilesanmi, Abayomi Akande, Aanuoluwapo Adeyimika Afolabi

**Affiliations:** 1 Department of Community Medicine, College of Medicine, University of Ibadan, Ibadan, Oyo State, Nigeria,; 2 Department of Community Medicine, University College Hospital, Ibadan, Oyo State, Nigeria

**Keywords:** COVID-19, herd immunity, vaccine, case fatality rate, COVID-19 deaths

## Abstract

The coronavirus infection (COVID-19) to date has no vaccine or effective treatment. Herd immunity offers indirect protection to susceptible members of the population. If the acquired immunity of a community rises above 67%, then a gradual decline in the number of incident cases is recorded. How many deaths would have occurred in the West African countries by the time at least 67% of our people are infected with the present case fatality rate (CFR)? The objective of this study was to develop a forecast of the number of COVID-19 deaths that would be recorded to attain herd immunity for each country in West-Africa. We predicted the numbers of deaths using publicly available demographic and COVID-19 data. To attain herd immunity in West Africa 5.2 million COVID-19 deaths would have occurred assuming the CFR is maintained at the current rates in the region. Attention should be focused on strategies that would limit the spread of infection and protect the most vulnerable population groups while the race to develop an effective vaccine should be hastened.

## Perspectives

The coronavirus infection (COVID-19) has been transmitted to more than 200 countries with nearly 7 million confirmed cases and 401, 970 deaths globally as at 8th June, 2020 [1]. In a completely susceptible population, a pathogen spreads rapidly in an unchecked manner following sufficient exposure to infected persons. However, if immunity exists among a significant proportion of the population, the likelihood of transmission of the pathogen will be reduced. Thus, the herd immunity threshold is defined as the level at which the proportion of susceptible individuals falls below the required threshold for transmission of the pathogen and varies across populations [2-4] Herd immunity offers an indirect protection to susceptible members of the population because of the existence of a large proportion of immune individuals present in the population. Such effects at the population level is considered in the design of vaccination programs which aims to attain herd immunity for the entire population, in such a way as to protect the vulnerable groups who may be susceptible to the disease [3,4].

The herd immunity simply depends on the basic reproduction number (R0) of the pathogen, and is defined as the average number of secondary infections introduced by a single infectious individual introduced into a wholly susceptible population [2]. Mathematically, the herd immunity threshold is defined by 1- (1/R0) (if for instance, R0 =3, the corresponding herd immunity threshold is 0.67) [2]. The Coronavirus has been estimated right from its onset to have a basic reproduction number (R0) between 2 and 6 [5], with differing values across countries. The differences in the R0 value is thus a reflection of the variation of the transmission dynamics of the SARS-COV-2 infection across countries. If the acquired immunity of a community rises above 67%, then a gradual decline in the number of incident cases is recorded. With direct impacts on the R0 and herd immunity threshold, the communicability of any infectious disease as in this case, SARS-COV-2 depends on factors such as: population structure, population density, and differences in the contact rates across existing demographic groups [4]. With the exposure of all persons including the susceptible and low-risk individuals to the viral infection, it is being rationalized that herd immunity could place at bay the current COVID-19 with which the entire globe is faced [6]. With an estimate of vaccine trial delaying for as long as 12-18 months, herd immunity against the SARS-COV-2 infection through natural infection is being sought as a possible remedy. Rather than the measures to prevent further spread of the Coronavirus pandemic such as the closure of schools and lockdown of entire cities across the World, an alternative strategy would be the exposure of all persons to the Coronavirus which in turn increases the population herd immunity to the viral infection.

Immunity against the Middle East Respiratory Syndrome (MERS) coronavirus by specific T-lymphocytes has been considered to offer up to 4 years protection [6]. Estimates from closely related Coronaviruses suggest that protective immunity could only be assured for 1 year, after which it might wane [5,6]. The question we are then posed with is this: How many deaths would have occurred in the West African countries by the time at least 67% of the population are infected at the present case fatality rate (CFR)? The objective of this study is to develop a forecast of the number of COVID-19 deaths that would be recorded to attain herd immunity for each country in West-Africa. We define inclusion criteria as a country within the West African subregion, having active transmission of COVID-19 reported in the country, and at least one death due to COVID-19 for the week ending 04-06-2020, the number of West African countries included based on these criteria is 15. We forecast the number of potential deaths as the reporting of deaths is likely to be more reliable and stable indicator of disease impact over time than the reporting of cases. Forecasts of deaths will help inform public health decision-making by projecting the likely number of deaths that would be recorded before herd immunity is attained.

In this study, the cumulative number of confirmed cases of COVID-19 and deaths were collected from publicly available data of the outbreak situation report of the World Health Organization (WHO) Coronavirus Disease (COVID-19) Dashboard on the 4th day of June 2020 [7,8]. The population of the country was obtained from the website of the population reference bureau. We predicted the numbers of deaths using demographic and COVID-19 data. Herd immunity is expected to be achieved at 67% infection rate. The mid-year population and country-specific CFR was used to calculate the expected deaths for each country if 67% were to be infected. (CFR of COVID-19 multiplied by the projected number of infections to have achieved herd immunity) Table 1 shows the population distribution in West Africa, cases, death, and case fatality rate of COVID-19. The country with the highest population in West Africa is Nigeria. Nigeria has a population of 201 million according to the world population bureau. As at 4th June 2020 Nigeria has had a total of 11,166 cases of COVID-19 and 315 deaths with a case fatality rate (CFR) of 2.8%. To have herd immunity to COVID-19 in Nigeria 134.7 million persons will have to be infected and 3.8 million deaths based on the present CFR of 2.8%. Cape Verde has the lowest population, 0.5 million in West Africa, 477 cases of COVID-19, five deaths and a CFR of 1.1%. To have herd immunity to COVID-19 in Cape Verde 402, 000 person will have to be infected and 4,422 deaths based on their present CFR of 1.1%. The overall CFR of COVID-19 is 2% in West Africa, and thus, at the present CFR 5.2 million deaths would have occurred before attaining herd immunity in the region.

**Table 1:** population distribution in West Africa countries, COVID-19 cases, deaths and case fatality rate, and projected number of infection and death to have herd immunity to COVID-19

Countries	Population mid 2019 in million	Cases	Death	Case Fatality Rate	Projected number of infections to have herd immunity (67%)	Projected number of deaths before herd immunity
Benin	11.8	244	3	1.3	7906000	102778
Burkina Faso	20.3	884	53	6	13601000	816060
Cape Verde	0.6	477	5	1.1	402000	4422
Côte d'Ivoire	25.5	3110	35	1.1	17085000	187935
The Gambia	2.3	26	1	3.8	1541000	58558
Ghana	30.3	8885	38	0.4	20301000	81204
Guinea	12.2	3933	23	0.6	8174000	49044
Guinea-Bissau	1.9	1346	9	0.7	1273000	8911
Liberia	4.9	316	28	8.9	3283000	292187
Mali	19.7	1386	79	5.7	13199000	752343
Niger	23.3	961	65	6.7	15611000	1045937
Nigeria	201	11166	315	2.8	134670000	3770760
Senegal	16.3	4021	50	1.4	10921000	152894
Sierra Leone	7.8	909	47	5.2	5226000	271752
Togo	8.1	452	13	2.9	5427000	157383
**Western Africa**	390	38116	764	2	261300000	5226000

Herd immunity would be attained when the projected number of COVID-19 deaths reaches 5.2 million assuming the CFR is maintained at the current rates for each country in the region. SARS-COV 2 is a novel pathogen and the dynamics of its infectivity and immunogenicity are still evolving. The numerous complexities that would ultimately determine disease spread and severity are still being evaluated. The extent to which humans can generate long-lasting protective immunity to SARS-COV-2 remains unclear. However, the unacceptably high projected COVID-19 deaths for herd immunity to occur is a clear indication that in the absence of strong public health interventions like a successful COVID-19 vaccine, establishing herd immunity by natural infection is not a wise strategy as the consequences could be devastating. Rather, attention should be focused on strategies that would limit the spread of infection and protect the most vulnerable population groups while the race to develop an effective vaccine should be hastened.
